# Understanding how to facilitate continence for people with dementia in acute hospital settings: a mixed methods systematic review and thematic synthesis

**DOI:** 10.1186/s13643-021-01743-0

**Published:** 2021-07-06

**Authors:** Deborah Edwards, Jane Harden, Aled Jones, Katie Featherstone

**Affiliations:** School of Healthcare Sciences, College of Biomedical and Life Sciences, Cardiff University, Eastgate House, 35-43 Newport Road, Cardiff, CF24 0AB UK

**Keywords:** Dementia, Continence, Communication, Individualised care, Systematic review

## Abstract

**Background:**

People living with dementia (PLWD) are at significant risk of developing urinary and/or faecal incontinence and are also at risk of functional incontinence or being labelled as being incontinent. Despite the growing population of PLWD and importance of continence care, little is known about the appropriate management, organisation, and interactional strategies for PLWD admitted to acute hospitals. This mixed methods systematic review and thematic synthesis sought to identify successful strategies across all care settings that could then be used to inform innovations in continence care for PLWD in the acute hospital setting.

**Methods:**

In phase 1, a scoping search of two electronic databases (MEDLINE and PsycINFO) and a consultation with stakeholders was undertaken. Findings were presented to the project steering group and two priority areas for phase 2 were identified which were communication and individualised care plans. In phase 2, eight databases and relevant UK government and other organisational websites were searched for English language citations from inception to August 2020. Critical appraisal was conducted using the Mixed Methods Appraisal Tool (MMAT Version 11). Thematic synthesis was employed and the strength of synthesised findings for the intervention studies was assessed using the GRADE approach and the confidence in synthesised qualitative and survey findings was assessed using the CERQual approach.

**Results:**

In phase 1, 1348 citations were found and 75 included. In phase 2, 6247 citations were found, 14 research studies and 14 policy and guidance documents were included. The quality of studies varied. Material was synthesised into three overarching syntheses which were: communication this is dignified, person-centred and respectful; communication during outpatients apointments and delivering individualised continence care.

**Conclusions:**

Recognising that PLWD are not always able to communicate their continence needs verbally is important. Incorporating interpersonal and communication skills into the context of continence care within training for those working with this patient group is crucial for continence to be maintained during an acute admission. Continence care in the acute setting should be tailored to the individual and be developed in partnership with staff and caregivers.

**Trial registration:**

PROSPERO: CRD42018119495.

**Supplementary Information:**

The online version contains supplementary material available at 10.1186/s13643-021-01743-0.

## Background

There are currently around 885,000 people living with dementia (PLWD) in the UK [1], and around 50 million people worldwide [2]. This number is likely to increase to over 2 million in the UK [3] and 13.5 million worldwide by 2050 [4]. People living with dementia are at significant risk of developing urinary and/or faecal incontinence [5] and are at risk of functional incontinence or being labelled as incontinent. Urinary incontinence (UI) is described as “the complaint of any in-voluntary leakage of urine” ([6], p. 1622) and is more common in older people. Faecal incontinence (FI) is defined as “the involuntary loss of liquid or solid stool that is a social or hygienic problem” ([7], p. 199)*.* The prevalence of FI is higher in PLWD compared to others of similar age [5, 8]. Functional incontinence on the other hand occurs when a usually continent person is unable to reach the toilet in time or as in PLWD, an inability to recognise the need to go to toilet, locate the toilet, or access the toilet [9], which is often a result of the environment they are in, rather than a feature of their dementia [10].

Dementia as a condition is often thought of as something initially cared for in the community, then later in specialist and long-term care settings, but the prominence of the acute hospital setting and its impact on PLWD cannot be ignored. The acute hospital setting has become a key site of care for PLWD. Internationally, prevalence estimates from a range of studies conducted since 2009 have reported that dementia was present in 18 to 42% of older adults admitted to hospital [11–15]. In the UK, the Department of Health in England acknowledges that at any given time, as many as one in four acute hospital beds will be occupied by a PLWD, who have been admitted with an additional acute condition [16, 17]. Although incontinence is recognised as a typical feature of advanced dementia, the majority of PLWD admitted to acute hospital wards with an unrelated condition are usually in the early and moderate stages of the disease, and thus, incontinence should not be a typical feature of their dementia [18]. Yet national acute audits conducted in the UK consistently identify PLWD and patients over 65 as being at high risk of being classified as incontinent and of receiving particularly poor continence care during acute admissions [19–21].

Studies have shown that a number of organisational factors within hospital environments that can contribute to the development of incontinence in PLWD; including lack of appropriate signage, insufficient privacy, poor orientation, lack of toilets, and use of continence aids [22, 23]. As a result, just over a third of hospitalised PLWD were reported to have developed UI at the time of discharge and, of those, 2% also developed FI for the first time [24].

Throughout the literature, nurses consistently report that ‘containment’ through the use of disposable continence pads and catheters as a key strategy for the management of continence for hospitalised older adults [25]. These approaches have implications for the occurrence of avoidable harm and patient outcomes during an acute admission; incontinence is a common risk factor for falls [26, 27], and catheters are associated with high rates of urinary tract infections [28]. These factors are also associated with a greater financial burden, prolonged hospitalisation, re-admission, and increased mortality [29–31]. Incontinence is recognised as potentially emotionally demeaning [32], humiliating, and embarrassing [33] for the person, and combined with dementia, increases the stigma [34] PLWD already experience [35–37], which can have significant negative impacts on quality of life [38].

Despite the growing population of PLWD and importance of continence care for this group [39], little is known about the appropriate management, organisation, and interactional strategies for PLWD admitted to acute hospitals [40]. Although several high-quality reviews have explored issues of continence for PLWD living at home [38, 41] and those living in longer-term care [8], only one previous review conducted just over 10 years ago examined incontinence care for PLWD across all care settings, focussing on assessment, management, and prevention [40]. On conducting a scoping search of the literature, we identified very little empirical research examining continence care for PLWD in acute settings. It was therefore decided to conduct a mixed methods systematic review and thematic synthesis across different care settings to identify successful strategies that could be used to inform innovations in continence care for PLWD in the acute hospital setting. This review was conducted as part of a wider ethnographic study that examined continence care, within the overall context of ward care in the acute setting for PLWD [42].

## Methods

### Design

This systematic review used the two-stage Evidence for Policy and Practice Information and Co-ordinating Centre (EPPI-Centre) approach [43, 44]. This involved a scoping of the overall area under review, followed by a targeted, in-depth, review and synthesis of the evidence in one or more sub-areas guided by key stakeholders in the field. The review combined quantitative, qualitative, and non-research material (e.g. policies and guidelines) and these strands were brought together into an overall thematic synthesis [45]. The reporting of this systematic review has been developed in accordance with the recommendations from the Preferred Reporting Items for Systematic Reviews and Meta-Analyses (PRISMA) statement [46]. The protocol has been registered in the International Prospective Register of Systematic Reviews (PROSPERO) (Registration: CRD42018119495).

### Scoping exercise

The first phase was a scoping exercise that asked “What is known about the management and practices of continence care (continence care, incontinence care, toileting, and catheter care) for PLWD in acute, long-term community healthcare, and home settings? Two databases were searched (MEDLINE and PsycINFO) from database inception to January 2018 for citations (a citation could be a research report, a review paper, a discussion piece, a published opinion, an editorial or something similar) that focused on, or contained an element relating to each of the following inclusion criteria:

1. People living with dementia, Alzheimer’s disease (AD) or cognitive impairment.

2. Acute, long-term, and community healthcare and home settings.

3. Urinary or faecal continence/incontinence, or toileting issues.

4. Conservative management or care practices (defined as “any therapy that does not involve pharmacological or surgical intervention” ([47], p. 1020) including catheterisation.

Of the 1348 citations retrieved, 87 were included (see Additional file S1 for flow of citations). The findings were summarised into a number of broad, descriptive, maps [48] to identify the ways in which continence is assessed and managed across settings. The findings from the scoping exercise in keeping with the EPPI-Centre approach were presented to stakeholders with interest in the field in order to ascertain views on the priority areas for the second phase of searching. The key stakeholder groups included PLWD, family carers, and practitioners drawn from different occupational groups (n = 32) and are shown in Table 1). All stakeholders as part of this process were asked to complete a priority setting exercise which was facilitated by answering the question. “What do you think are five of the most important ways that continence could be managed for PLWD when they are in hospital?” The responses from the individual and group consultations were collated, coded and grouped together and a list of the ways of managing continence in the hospital setting was generated.

**Table 1:** Table of stakeholders who took part in the consultation exercise

Stakeholders	Source of contact	Source of information
DCAs Young onset team DCA (n = 1) REACT crisis team DCA (n = 1) (when a person has an additional mental health crisis on top of their dementia) Community DCA’s (n = 3)	SOLACE A service within the local University Health Board which exists to provide support to carers and those diagnosed with dementia, depression or severe later life mental illness. Their aim to help prevent admission to hospital and deterioration in relation to being in hospital.	Group discussion followed by individual priority setting exercise
DCA’s	Liaison Psychiatry A service that covers wards in the general hospital setting. Their role is to help PLWD when they are in hospital if they are struggling and who are exhibiting behaviours that challenge or if they are anxious or agitated such as walking around a lot and the staff are not able to cope	Group interview followed by individual priority setting exercise
Continence service team Nurse consultant (n = 1) CNSs (n = 7)	NHS Continence Service An outpatient based service. The role of the team is to accept and take referrals from primary care general practitioners district nurses and others to see patients with incontinence and to assess and put in place a suitable management plan for them	Group discussion followed by individual priority setting exercise with CNSs Individual Interview with nurse consultant followed by setting exercise
Occupational therapist (n = 1)	Facebook Currently works on an elderly ward with both functional patients and PLWD. Previous employment was on a specific dementia ward in a community hospital	Individual interview followed by priority setting exercise
PLWD (n = 2) Family carers (n = 11) DCA (n = 10) Activities coordinator of local care home (n = 1) Volunteer from the Alzheimer’s society (n = 1)	Dementia Consultation Event A whole day event in which issues around toileting and continence were explored through narrative and creative presentations (through pictures, poems and artistic expression, arts, and discussion.	Group discussion followed by individual priority setting exercise

Key: *CNS*: clinical nurse specialists, *DCA* dementia care advisors, *PLWD* people living with dementia

Descriptive maps of the findings from phase 1 and a summary of the consultation with the stakeholders were presented to the collaborative research/project team of co-applicants which included two family carers, the director of research and development at an NHS Health Board, and six researchers (from the disciplines of sociology, nursing, social policy, anthropology). Informed by the principles of nominal group technique [49, 50], those present were invited to record on a Post-it note written responses to the question ‘What do you think are the most important ways that continence could be managed for PLWD when they are in hospital. After the meeting, items were coded and grouped together, and a list of ranked priority risk categories was created and circulated to the group for approval. The top 2 priority areas identified as having the most relevance to informing and improving continence care within the acute setting across both groups was ‘communication’ and ‘individualised care planning’, which were taken forward for the second in-depth phase of the review. This exercise informed the research question that was taken forward to the mixed-methods review exercise, which was “What is known about the management and practices of continence care in relation to communication and individualised care planning for PLWD a in acute, long-term community healthcare, and home settings?”

### Objectives

1. To explore carers’, family members’, and health care professionals’ (HCPs) perceptions and experiences of communication and individualised care planning for PLWD with regard to toileting and continence.
2. To identify the communication strategies and the use of individualised care planning employed by carers’, family members, and HCPs to manage toileting and continence for PLWD.

### Eligibility criteria

We used PICOS/PICo framework to guide the inclusion criteria on participants (P), intervention/phenomena of interest (I), comparators (C), outcome (O), study design (S), and context (Co)

#### Participants

PLWD or cognitive impairment and/or carers’, family members’, and HCPs of PLWD or cognitive impairment. All dementia subtypes were included for example AD, vascular dementia, and frontotemporal dementia.

#### Interventions/phenomena of interest

Any communication strategy or individualised care plan/s that carers, family members, and HCPs have employed to manage toileting and continence for PLWD.

Perceptions and experiences of communication and/or individualised care planning for PLWD with regard to toileting and continence.

#### Comparators

All comparisons were considered.

#### Outcomes

All outcomes as presented across the primary studies that related to communication and individualised care planning.

#### Study designs

Quantitative (e.g. randomised controlled trials (RCTs), quasi-experimental, cohort studies, descriptive studies), qualitative studies (e.g. focus groups or individual interviews), and non-research material (e.g. policies (UK only), guidelines, reports of practice initiatives, and clinical case studies).

#### Context

A PLWD and all those involved in their care in acute, long-term, and community healthcare and home settings.

### Searching

Searches were made for English language citations using the following eight databases, with time limits from database inception to June 2018 (updated August 2020). On the Ovid Platform: MEDLINE: PsycINFO; EMBASE, on the EBSCO Platform: CINAHL, ERIC; on the ProQuest platform; ASSIA and Open Grey. Relevant UK government and organisational websites (for example Alzheimer’s Society and Dementia UK) were searched. Keywords and index terms identified as relevant and reflecting the projects agreed priorities in phase 1 were used and individual search strategies developed for each database. This review also drew on the individual search strategies developed for the Cochrane Incontinence Review Group [51]. An example search strategy for MEDLINE is provided in Additional file S2.

To identify published resources that have not yet been catalogued in the electronic databases, recent editions of the Journal of Gerontological Nursing, American Journal of Alzheimer’s Disease & Other Dementia, Journal of the American Geriatrics Society and the Journal of Wound, Ostomy, and Continence Nursing were hand-searched. Reference lists of included studies were scanned, experts contacted, and forward citation tracking performed using ISI Web of Science.

### Screening, quality appraisal, and data extraction

Screening and selection of all citations was conducted using standardised systematic review methods involving all members of the project team [52]. Multiple articles by the same authors reporting findings from the same study were linked together to help inform decisions on which studies to include. The methodological quality of all included research publications were independently assessed by two reviewers using Mixed Methods Appraisal Tool (MMAT-Version 2011). This tool was developed for the appraisal of methodological quality of qualitative, quantitative, and mixed methods studies [53, 54]. Any disagreement on quality was resolved through discussion with a third reviewer. Each study was assigned a score based on the number of criteria met (25%—one criterion met; 100%—all criteria met). Studies were excluded from the review if they scored less than 50% for quality, meaning that they fulfilled a maximum of only two of four criteria [53]. Non-research evidence (e.g. policies, reports) were not subjected to quality appraisal. For the purposes of this review, the study findings for each primary research study were considered to be all text that was labelled within each publication as results or findings. All non-research materials were available as electronic documents and were searched using keywords relevant to the priority areas (for example communication, tailored, individual). This data were then considered to be findings and extracted and entered verbatim into Microsoft WORD (see Additional file S3).

### Synthesis

Thematic synthesis was employed to bring together data from both qualitative and quantitative primary research studies and non-research material [45]. The full text of all quantitative and qualitative research studies along with relevant extracts (communication and individualised care planning) from included policies and guidance were uploaded into NVIVO-12^TM^ [https://www.qsrinternational.com/nvivo-qualitative-data-analysis-software/home]. For the qualitative studies, codes were generated through line-by-line coding of text of the findings and a coding frame developed. This process was carried out inductively based on close reading by one reviewer of the content of all items and subsequently checked by a second reviewer with any disagreements resolved through discussion. Next, the quantitative data was ‘qualitised’ in which the quantitative data was converted into textual descriptions to allow integration with the qualitative data [55]. For this process, the descriptive codes were used to categorise the text of the findings from the quantitative studies. This process was also followed for the extracted data from the policy and guidance documents. All of the codes were then grouped into descriptive themes that captured and described patterns across all the data. Once this process had been completed, the next step was to create analytical themes so that findings could be synthesised across all the studies and non-research material and their collective meaning interpreted in relation to our review objectives [44].

The confidence of the overarching synthesised findings derived from descriptive quantitative (that had undergone qualitisation) and qualitative research was assessed using the Confidence in the Evidence from Reviews of Qualitative research (CERQual) approach [56] and the findings from quantitative experimental research was assessed using the Grading of Recommendations Assessment, Development, and Evaluation (GRADE) approach [57]. The original CERQual approach was designed for qualitative findings but has previously been used by members of this research team (DE) in additionally adopting CERQual for the assessment of the confidence of synthesised findings from descriptive quantitative studies that have undergone qualitisation [58–60]. The confidence of synthesised review findings is based on the assessment of four components: the methodological limitations of the qualitative studies contributing to a synthesised review finding, the relevance to the review question of the studies contributing to a synthesised review finding, the coherence of a synthesised review finding, and the adequacy of data supporting a synthesised review finding. Four levels are then used to describe the overall assessment of confidence as high, moderate, low, or very low. When a synthesised review finding is assessed as being ‘high confidence’, this indicates that this synthesised review finding should be seen as a reasonable representation of the phenomenon of interest. If there are concerns with regard to any of the above four components, then this indication is weakened, and a lower level of confidence attained [56]. The GRADE approach rates the quality of a body of evidence as high (further research is very unlikely to change our confidence in the estimate of effect), moderate (further research is likely to have an important impact on our confidence in the estimate of effect and may change the estimate), low (further research is very likely to have an important impact on our confidence in the estimate of effect and is likely to change the estimate), or very low (any estimate of effect is very uncertain).

## Results

### Description of included material

**Fig. 1:**
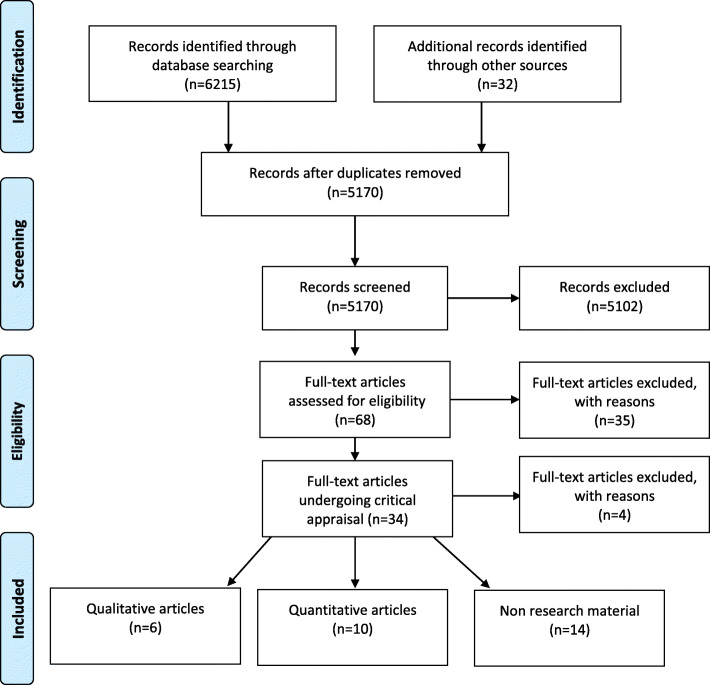
Flow of studies through review

The database searches yielded a total of 6247 citations after duplicates were removed (see Fig. 1). Sixteen research publications (consisting of 15 unique research studies) were included in the final review along with a total of 14 policy and guidance documents. Details of full-text publications excluded from the review are provided in Additional file S4.

### Characteristics of the included material

Information on the characteristics of included research studies, including assessments of quality, are given in Tables 2, 3, and 4 and details of the non-research material is available in Additional file S4.

**Table 2:** Characteristics of included qualitative studies

Author/s, year, country Aim	Setting Participants	Demographic details for PLWD	Methods MMAT score
Study 1: Bliss et al. 2013 [61] USA To describe health literacy needs related to incontinence and skin care among family or friend caregivers of individuals with AD and develop supportive and educational materials that address these	Setting Home Participants Family/friend adult caregivers (n = 48) Spouses (44%), daughters (31%), or extended family members/friends (25%) Recruited from community-based agencies	Gender Female (75%) Age (mean + SD) years 64 ± 14 Mental status AD or dementia	Methods Focus groups and interviews MMAT score: 100%
Study 1: Mullins et al. 2016 [62] USA To examine barriers to communicating with healthcare professionals and health literacy about incontinence among different types of informal caregivers of individuals with AD	Same as Bliss et al. 2013	See Bliss et al. 2013	See Bliss et al. 2013 MMAT score: 100%
Study 2: Hutchinson et al. 1996 [63] USA To addresses the range and variation of toileting problems, management strategies used by family and employed caregivers	Setting AD specific day centre Home Participants Family members who participated in the centre support groups (n = 16) Staff members employed at the day care centre (n = 13)	Demographic characteristics of patients with AD who attended the day centre were not reported Mental status AD	Methods Participant observation at the day care centre, clients’ home, and support groups Interviews with families and staff members Based on qualitative ethology MMAT score: 75%
Study 3: Rolnick et al. 2013 [64] USA To examine healthcare providers’ perspectives regarding improving communication with patients and their caregivers about incontinence and dementia	Setting Secondary care providers Participants Physicians (n = 8)/nurse practitioners (n = 2)/pharmacist (n = 1) Potential participants suggested by advisory committee	Not applicable Mental status Dementia	Methods Interviews MMAT score: 100%
Study 4: Ostaszkiewicz et al. 2018 [65] Australia To explore nursing home staff members’ beliefs and expectations about what constitutes “quality continence care” for people living in nursing homes	Setting Nursing home Participants Nursing home staff (n = 19) Registered nurses (n = 8) Enrolled nurses (n = 4) Personal care workers (n = 7) Recruited using snowballing technique; selective placement of information in print and electronic media; and information sessions at several nursing homes	Not applicable Mental status Most nursing home residents were cognitively impaired	Methods Interviews Naturalistic inquiry using a qualitative exploratory descriptive research approach MMAT score: 100%
Study 14: Scerri et al. 2018 [66] Malta To categorise the perceived and observed needs of persons with dementia admitted in acute medical wards and to explore whether these needs are being or have been met.	Setting Acute medical wards (n = 3) Participants PLWD and their family members (n = 12)	Gender Age (mean) years 84.7 Range 71 to 93 Mental status Dementia	Methods Interviews Observations using dementia care mapping MMAT score: 75%

Key: *AD* Alzheimer’s disease, *MMAT* Mixed Methods Appraisal Tool, *PLWD* people living with dementia, *SD* standard deviation

**Table 3:** Characteristics of included descriptive studies

Author/s, year, country Aim	Setting Participants	Demographic details for PLWD	Data collection Outcome measures MMAT score
**Cross sectional surveys**			
Study 7: Wilkinson et al. 1995 [67] Australia To evaluate the comparative suitability of a range of words or symbols to label a toilet for people with dementia	Setting Phase 1: Hostel care for ambulant people with dementia (n = 24/28, rr 86%) Phase 2: Aged care complex with hostel and nursing home facilities (n = 28) and an acute hospital ward (n = 20) Participants Phase 1: n = 24 institutions Phase 2: n = 24 patients	Gender No details provided Age (years) 80.4 (95% CI 77.1–83.1) Mental status Folstein MSE Normal cognition (n = 21) Mild dementia (n = 11) Moderate dementia (n = 16) Severe dementia excluded The study comprised two phases and questionnaires were used in both	Data collection Phase 1: questions posed to hostel management on what word and/or symbols were already in use in that institution to label toilet and/or bathroom facilities Phase 2: questions asking preference for toilet door labelling Outcome measures Preferred symbol according to cognitive state Preferred word according to cognitive state MMAT score: 100%
Study 12: Shih et al. 2015 [68] Taiwan To understand and compare the behavioural characteristics of bowel movement and urination needs in patients with dementia	Setting Long-term care facilities (n = 8) Day centre (n = 1) Participants Residents (n = 187)	Gender: female (59%) Age (mean + SD) years 80.1 + 9.6/range 70 to 90 Mental status AD 38.5% Unspecified dementia 32.6% Vascular dementia 18.7% Other dementia 10.2%	Data collection Behaviour checklist for bowel and urination developed for the study Outcomes measures Symptom’s and signs of bowel movement and urination expressed by the patient MMAT score: 100%
**An adapted three-stage Delphi consultation study**			
Study 13: Iliffe et al. 2015 [69] UK Phase 4 The aim of this study was to develop and test a continence assessment tool and supporting resources for people with dementia, to be used by primary care professionals, primarily community nurses (p. 95)	Setting Community Participants Stage 1 Carers and professionals (n = 10) Stage 2 Carers and professionals (n = 10) Specialist continence professionals (n = 10) Stage 3 Carers (n = 8) General Practitioner (n = 2), Geriatrician/psychogeriatrician (n = 1) Continence nurse specialist (n = 3) District nurse/community nurse (n = 7) Occupational therapist (n = 2) Other (n = 3) (rr = 26/50)	Not applicable Mental status Dementia	Data Collection Stage 1: Face to face consultations were facilitated to describe a broad range of principles and issues that would underpin an assessment tool designed to address the needs of people with dementia Stage 2: A prototype dementia-focused continence assessment tool was developed using the data generated in stage 1, asking for agreement or disagreement to items plus suggestions for further items. This was used to consult, in writing, both the expert group in stage 1 and also a further group of carers and specialist continence professionals. The prototype was further adapted. Stage 3: A different, wider group of experts (carers and professionals) was consulted in writing. They were sent the draft dementia-focused assessment tool together with a questionnaire to test its face and content validity. Outcome measures Recipients were asked (1) whether or not the tool would improve recognition of the problems (face validity) and (b) to rate each item for importance and identify missing or unnecessary items (content validity) MMAT score: 75%

Key: *AD* Alzheimer’s disease, *CI* confidence intervals, *MMAT* Mixed Methods Appraisal Tool, *MSE* Mental State Examination, *PLWD* people living with dementia, *SD* standard deviation

**Table 4:** Characteristics of included quantitative experimental studies

Author/s, year Country Aim	Setting Participants Demographic details for PLWD	Intervention	Data collection Outcome measures MMAT score
**Case series with non-concurrent multiple baseline design**			
Study 5: Lancioni et al. 2009a [70] USA The authors presented three pilot studies that assessed the effectiveness of verbal instructions, presented automatically through simple technology, in helping persons with mild-to-moderate AD recapture basic daily activities	Setting Alzheimer rehabilitation centre Participants Residents with AD (n = 3) Gender: Female (100%) Age (years): 79, 81, 86 Mental status AD MMSE scores: 10, 19, 22	Intervention Baseline: Pilot study 1: the participants were to perform the bathroom routine without the help of the technology and related verbal instructions Intervention: pilot study 1: The participants performed all bathroom-routine steps with the help of the technology, which presented the instructions Step 1 was “sit on the toilet”. 17 steps in total and step 1 was “to sit on the toilet”	Data collection The participants’ performance of a step was recorded as ‘correct’ if it matched the description of such step (and the instruction available for it during the intervention) and occurred independent of prompting by research assistants Outcome measures Percentage of correct steps performed MMAT score: 100%
Study 6: Lancioni et al. 2009 [71] USA To assess the effectiveness of verbal instructions (presented automatically through simple technology) in helping persons with mild or moderate AD perform daily living activities	Setting Alzheimer rehabilitation centre Participants Residents with AD (n = 4) Gender: female (100%) Age (years): 59, 76, 79, 85 Mental status AD MMSE scores: 11, 12, 16, 20	Intervention Same as Lancioni et al. 2009a Four studies with the first one aimed at replicating pilot study 1 from Lancioni et al. 2009a. efforts directed at re-establishing the performance of morning bathroom routine	Data collection Same as Lancioni et al. 2009a Outcome measures Same as Lancioni et al. 2009a MMAT score: 100%
**Randomised control trials**			
Study 8: Jirovec and Templin 2001 [72] USA To evaluate the effectiveness of an individualised scheduled toileting program on incontinent, memory impaired elders being cared for at home	Setting: home Participants Caregivers (n = 118) Memory impaired elders (n = 118) Randomised to I (n = 77), C (n = 41) Recruited through announcements in newsletters, flyers on bulletin boards, and newspaper advertisements asking for volunteers who were caring for a memory-impaired elder Gender: female (69%) Age (mean + SD) years 79.89 + 7.93 Mental status SPMSQ: mean 6.69 + 2.28	Intervention individualised scheduled toileting program The intervention group was taught an IST procedure that compensated for cognitive impairment by providing memory-impaired patients toileting reminders Initially, assignment was to one of two intervention groups: one group of participants was visited every 2 months, and the other group after a 6-month interval. There was also a control group At the 6-month follow-up, the two intervention groups did not differ with respect to UI. The original two intervention groups were combined, leaving a single intervention group and a control group.	Data collection Incontinence was calculated as the percentage of time the patient was incontinent by dividing the incontinent episodes by the total number of voiding episodes, both continent and incontinent Voiding record Outcome measures Decrease in percentage of incontinent episodes versus staying the same or not showing improvement in incontinence Incontinence frequency Mobility Consistency in implementing the IST protocol MMAT score: 75%
**Prospective cohort study**			
Study 15: Wijk et al. 2018 [73] Sweden To operationalise, assess, and evaluate the feasibility and preliminary effects of implementing a person-centres approach to incontinence care for older adults with cognitive decline in residential care facilities in Sweden	Setting Residential care facilities (n = 3) Participants Health care workers (n = 20) Residents with cognitive decline (n = 54) Gender Female (59.9%) Age (mean + SD) years 83.9 + 8.72 Range 68 to 99 Mental status Cognitive decline MMSE score of 9.28 + 7.94	Intervention Person centred approach focused on assessment and care planning to incontinence care over a 10-month period Training was provided over 5 session s to teach participants how to tailor a person-centred incontinence plan At the end of the 10-month period the participants created guidelines to make change towards person-centred incontinence care sustainable	Data collection Health care records assessed by research team at baseline, immediately after and at 6 months Process outcome measures of the person-centred approach Impact outcome measures of participants quality of life Impact outcome measures of participants quality of care Outcome measures Quality of life in late stage dementia Continence status (totally independent—using the toilet with no need of any containment product; partly continent—continent if assisted when needing to go to the toilet with or without use of a containment product; totally incontinent—being dependent on containment products 24/7 and not managing by oneself Has baseline assessment of incontinence been conducted? Have person centred actions been taken regarding incontinence? Has the resident been given adapted continence aids? MMAT score 75%
**Pre-test/post-test**			
Study 9: Tanaka et al. 2009 [74] Japan To investigate whether a system of individualised and comprehensive care was able to increase the intake of fluids and food, and to reduce the proportion of diaper users and the size of their diaper pads, thus leading to an enhanced quality of life	Setting Nursing homes (n = 17) Participants Nursing home residents (n = 122) Gender Female (85.2%) Age (mean) years 85.2 Mental status Dementia	Intervention Individualised and comprehensive care that focused on providing adequate fluids and meals, encouraging patients to use toilets and reducing the size of their diaper pads. This approach would differ significantly from the usual UI care in which diapers would be changed only at scheduled times	Data collection methods Water intake volume, condition of diapers (dry or wet), when residents wet their diapers were recorded in residents check sheets by staff Hours spent in wet diapers were calculated by subtracting the total time spent in dry diapers from 24 h Types of pants or diapers (cloth pants, training pants, diaper, cloth diapers), and the size of pads (S, M, L, XL, 2XL) Method of daytime urination (toilet, commode chair, urinary chamber pot, diaper Outcome measures Mean water intake volume Time spent in wet diapers (hours/day) Changing types of pants or diapers and the size of pads during daytime Change in method of daytime night-time urination MMAT score: 100%
**Post-intervention descriptive surveys**			
Study 10: Gitlin and Corcoran 1993 [75] USA To describe the use of the home environment by 17 spouse caregivers to manage problems associated with bathing and incontinence	Setting: Home Participants Spouse caregivers of elderly with dementia (n = 17) Recruited from a network of local social services agencies Demographic characteristics of elderly PLWD not provided Mental status Physician’s diagnosis of dementia	Intervention Individual treatment strategies delivered by an OT and designed to enhance the caregiver’s ability to problem solve about their environment and to develop effective solutions to situations they considered problematic	Data collection Data recording form completed by OT Outcome measures Number of solutions which were implemented by a caregiver Number of solutions deemed ineffective and which were eliminated by the caregiver MMAT score: 75%
Study 11: Corcoran and Gitlin 2001 [75] USA To describe the specific aspects of treatment that were accepted and utilised by 100 family caregivers	Setting: home Participants Family caregivers in the treatment arm of a RCT (n = 100) Recruited using media announcements and social service referrals Demographic characteristics of elderly PLWD not provided Mental status Physician’s diagnosis of dementia	Intervention Environmental Skill-Building Program Home environment intervention delivered by OTs and included toileting and incontinence same as Gitlin and Corcoran 1993	Data collection Interviews to ascertain: The specific problems areas that were addressed in the intervention The specific strategies that the caregiver indicated a willingness to try (attempted) The strategies the caregiver actually used Outcome measures Number and type of problem area Strategies for specific problems Strategies by environmental layers Acceptance and use of environmental strategies MMAT score: 75%

Key: *AD* Alzheimer’s disease, *CI* confidence intervals, *C* control, *I* intervention, *IST* individualised scheduled toileting, *MMAT* Mixed Methods Evaluation Tool, *MSE* Mental State Examination, *OT* occupational therapist, *PLWD* people living with dementia, *RCT* randomised controlled trial, *RR* response rate, *SPMSQ* Short Portable Mental Status Questionnaire, *UI* urinary incontinence

The research studies used a variety of research methodologies which included case series with non-concurrent multiple baselines (n = 2) [70, 71], RCT [72], pre-test/post-test [74], prospective cohort study [73], post-intervention descriptive surveys (n = 2) [75, 76]; cross-sectional survey (n = 2) [67, 68], an adapted three-stage Delphi consultation study [69] and qualitative methods (n = 5, across 6 publications) [61–66]. The non-research material consisted of web pages/web booklets (n = 5) [77–81], guidelines (n = 2) [10, 82], reports (n = 2) [83, 84], guidelines/guidance (n = 2) [33, 85], framework (n = 1) [86], model (n = 1) [87], and information sheets (n = 1) [88]. Eight research studies (across nine publications) were conducted in the USA [61–64, 70–72, 75, 76], two in Australia [65, 67], and one in each of the following countries: UK [69], Japan [74], Taiwan [68], Malta [66], and Sweden [73]. Only four of the non-research materials were published outside of the UK with one European guideline [10], one international guideline [33], and an Australian framework and the model published by the same author [86, 87]. The research studies were conducted across a variety of settings which included the home care and community setting (n = 5) [61, 69, 72, 75, 76], nursing homes (n = 2) [65, 74], a residential care facilit y [73], AD rehabilitation centres (n = 2) [70, 71], secondary care settings (n = 3) [64, 66, 68], residential treatment facility [73], and across multiple locations (AD specific day centre and home care setting [63] or hostel care for ambulant people with dementia, aged care complex with hostel and nursing home facilities, and an acute hospital ward [67] or a day centre and long-term care facility (LTCF) [68]). Across studies participants included PLWD [66, 67], residents of nursing homes who had a diagnosis of AD [70, 71], residents of LTCFs with cognitive decline [68], family members or caregivers of PLWD [61, 63, 66, 69, 72, 75, 76], day centre staff [63], care centre managers [67], nursing home staff [65], primary care providers [69], and secondary care providers [69]. Rolnick et al. conducted their study with a number of secondary care providers, and these were physicians, nurse practitioners, and pharmacists [64].

### Quality assessment of included research studies

The overall quality across the studies was variable. Two of the four qualitative studies fulfilled all four quality criteria on the MMAT, with the remaining two studies fulfilling three of the quality criteria but did not report whether the researcher’s role might influence the outcome of the study [63, 66]. The RCT fulfilled three out of the four quality criteria, with the complete outcome data (80% or above) not reported [72]. There were six quantitative non-randomised studies and of these three fulfilled all four quality criteria [70, 71, 74]; for two studies, it was not possible to ascertain the response rate for the sample [75, 76] and the other did not compare the baseline characteristics between those in the control and intervention groups [73]. The remaining three studies were quantitative descriptive, two studies fulfilled all four criteria [67, 68], and for the study that did not, we were unable to ascertain the response rate for the sample [69].

### Thematic synthesis

The findings from the quantitative and qualitative research, and from the included policy and guidance materials, were synthesised and three themes were created which were (a) communication that is dignified, person-centred, and respectful; (b) communication during outpatient appointments, and (c) delivering individualised continence care. These themes and the associated sub-themes are further discussed below.

### Theme 1: Communication that is dignified, person-centred, and respectful

Six of the included studies (across seven publications) [63–67, 70, 71] and six of the non-research publications [10, 79, 80, 82, 86, 87] reported findings related to theme 1. Six sub-themes were identified which included communicating in a dignified way; attitudes of HCPs towards continence and continence care; the importance of non-verbal cues; finding the appropriate words and symbols to describe the toilet, strategies for improving communication; and using technology to present instructions. Some aspects of these themes inevitably overlap as they are all in some way related to communication.

#### Communicating in a dignified way

The importance of protecting personal and social dignity [63–65] during continence care was significant and HCPs reported a belief that PLWD and their caregivers prefer not to talk about incontinence because it is a highly embarrassing [64, 65] and distressing issue [87]. Health care professionals believed that the provision of quality continence care for PLWD includes measures and approaches that conceal incontinence by creating situations that allowed PLWD to go to the toilet in private and avoiding communication, which revealed their issues around incontinence or care dependence that could cause them to feel embarrassed, ashamed, or humiliated [65].

The importance of respecting PLWDs right to privacy was also considered important [63, 65, 87]. In order to relieve PLWD perceived embarrassment of accepting assistance [63, 65], HCPs stressed the importance of building rapport and trust, using humour [87], and “acting natural” ([63], p. 24) when supporting continence needs. Health care professionals also felt that in order to communicate with PLWD in ways that would minimise any emotional impact that HCPs should have the appropriate knowledge and skills [65]. Other strategies to enhance privacy included whispering to the client about toileting issues [65] and keeping these issues secret [63]. However, HCPs acknowledged that PLWD may have difficulties in recognising and communicating their continence needs and that not being verbally able to request toileting assistance was viewed as a barrier to protecting dignity [65]. Closely overlapping with this theme of communication is the issue of HCPs attitudes towards continence care.

#### The attitudes of HCPs towards continence and continence care

The language used within a care environment is important regarding continence care [83, 86] and was identified as not always respectful [83] but where staff had good knowledge of the people they cared for, then these approaches were respectful and built good relationships with PLWD [83]. Ostaszkiewicz et al. [86] on discussing coercive continence care practices, described them as including the use of verbal or physical force to wash a person, to accept wearing continence pads or other forms of incontinence containment and to accept continence checks ([86] p. 2). The authors also suggest that chastising a person for being incontinent could be said to be a form of verbal abuse. Although some ward staff promote continence, this does not appear to happen consistently within acute settings [66]. Relatives expressed concern that PLWD would be happy to go to the toilet if assistance was provided, but that staff encouraged them to “do it in the nappy” ([66] p. 8).^.^ Other times, it was found that in some cases, routine toileting was avoided, and cues ignored when staff members were busy, or appeared uncomfortable with or disinterested in providing support [63, 66]. Ostaszkiewicz [86] emphasises that “Communicating therapeutically about incontinence with any person, including people with dementia, involves the demonstration of warmth, compassion and humanity” ([86], p. 523)*.* This is a skill that requires both clinical knowledge and interpersonal and communication skills, which should all be included within education programs [87]. Both formal caregivers and family carers would benefit from such programs, which would also enable the development of “empathetic understanding” ([86] p. 8) to the emotions that a PLWD has in response to incontinence and its care [86].

#### The importance of non-verbal cues

People living with dementia are not always able to recognise and communicate that they need to go to the toilet or indicate that they need assistance [10, 61, 63–65, 67, 70, 71, 75, 76, 80, 81, 83]. It is therefore important to recognise the non-verbal signals, body language, facial expressions, behaviours, and any signs that the PLWD uses to communicate in such instances [63, 80, 81, 83] so that their wishes can be acknowledged [83]. Listening carefully to the words or phrases that PLWD use for describing the toilet [67, 79, 81–83] as well as being able to recognise familiar gestures [67, 82, 83] is seen as important. New staff should be trained to recognise the importance of toileting and to how to understand individual behaviours and non-verbal cues in relation to toileting [63].

A range of different non-verbal cues had been observed or reported and included the following: someone pulling/taking off their clothing when they need to go to the toilet [10, 68, 80]; making particular sounds such as moaning or grunting [63, 68, 80]; assuming a different posture [10]; someone looking around [63]; fidgeting [10, 63, 79, 88]; getting up and walking around or pacing [63, 78, 79, 88] or restlessness [10, 68]; holding their crotch or their stomach [10, 63, 79]; different facial expressions such as worry [10] or sorrow [68]; going to the corner of the room [79] and pulling at their clothes [10, 88].

Hutchinson et al. also reported a number of affective cues which included anger, profanity, and “appearing frustrated and irritable” ([63] p. 21). Another study investigated common behaviours when PLWD experience either bowel movement or urination needs and found that anxiety, restlessness, and taking off/putting on clothes “inappropriately” occurred in more than 30% of patients [68].

#### Finding the appropriate words and symbols to describe the toilet

Wilkinson et al. sought to evaluate the comparative suitability of a range of words or symbols to label a toilet for PLWD. As part of an institutional survey (n = 24), the words that were used to label the toilet were “toilet” (67%), “male/female” (11%) and in some institutions, there was no labelling (22%) ([67] p. 163). Only four institutions used symbols, and these included the international symbol (n = 1), toilet symbol (n = 1), yellow wrapping over door (n = 1), and ceramic plaque upon which was written the word “toilet” ([67] p. 164). A further survey was conducted with PLWD and reported within the same publication and it was reported that the preferred word and symbol for toilet varied significantly (p < 0.05) according to their stage of dementia (which had been assessed using the Folstein Mental State Examination and classified as normal, mild, moderate, and advanced). “Ladies” and “gents” was preferred by those assessed with no cognitive impairment and “toilet” by those assessed with moderate dementia ([67] p. 164). The international symbol (male and female symbols) was preferred by people assessed with no cognitive impairment or mild dementia whilst the toilet symbol was preferred by those identified with more advanced dementia [67].

#### Strategies for improving communication

A number of general communication strategies for improving communication between HCPs and PLWD have been suggested. In order to reduce anxiety/fear/embarrassment, it is identified as being important to check HCPs awareness of good communication techniques when working with PLWD [69] and that HCPs introduce themselves and seek the PLWD’s approval before performing tasks [65]. Other suggestions include prompting [10, 80, 82, 83]; getting to know the PLWD [80], how they communicate [81] and determining their routines, habits, and lifestyle [79, 81]; getting HCPs to ask the PLWD how they can be help them manage their continence [79]; and communicating with the family to determine usual behaviour patterns [63] and not making assumptions and see the person as an individual [81]. One study described how nursing staff communicated with residents’ families about methods to manage incontinence when taking the PLWD “on an outing” ([65] p. 2432)*.* The advice included information about how to check and change continence pads, how to assist the resident to the toilet, and how long continence pads could potentially last without needing to be changed [65]. In another study, caregivers reported that they sought additional information about incontinence from the internet but were concerned about the accuracy of the information retrieved, whether they could understand it and had concerns about their searching skills [62]. They wanted support and reassurance from HCPs that they were providing the care that was required and they wanted information before any problems such as incontinence occurred so that they could feel prepared [61].

#### Using technology to present instructions

Two pilot studies [70, 71] conducted by the same authors explored the effectiveness of verbal instructions, presented automatically through simple technology, in helping people with mild-to-moderate AD regain basic daily activities. The technology consisted of a modified Walkman with recordings of verbal instructions that directed the PLWD to undertake bathroom-related activities in a certain order. Sensors detected when a PLWD entered the bathroom prompting the first instruction telling them to sit on the toilet. After a long pre-determined interval, this instruction was then followed by another instruction for them to wash their hands with the soap. The Walkman was activated by a battery-powered, radio-frequency photocell, light-reflecting paper, and a microprocessor-based electronic control unit. Data from both studies showed that the use of verbal instructions and basic technology to control their presentation has the potential to be effective in helping people with mild or moderate AD recapture relevant daily activities, including toileting [70, 71].

### Theme 2: Communication during outpatient appointments

Two of the included studies (across three publications) [61, 62, 64] reported findings related to theme 2. Four sub-themes were identified which included presence of PLWD during outpatient consultations; initiating conversations during outpatient consultations; the language of incontinence during outpatient consultations and resources for improving communication.

#### Presence of PLWD during outpatient consultations

There is a lack of consensus as to whether PLWD should be present with their caregivers during outpatient consultations [61, 62, 64]. Health care providers believed that care recipients should be present when discussing continence problems during consultations [64]; however, caregivers expressed mixed opinions [61, 62]. Caregivers, who favour this approach, view the HCP as an authority in this subject, with the result that they believe the PLWD would be more likely to cooperate with management strategies because they had been involved in the discussion [61]. Whereas those who opposed this reported that they did not want to upset or make their care recipient anxious by discussing a problem that the PLWD might not fully understand or be able to control [61]. Those caregivers, who were daughters, felt the need to be sensitive to their parent’s privacy and feelings, preferring to discuss incontinence in greater depth with their HCPs; this finding did not reflect spouses’ views. However, time constraints or inability to meet alone with the HCPs prevented in-depth discussions from taking place [62]. Some caregivers suggested that HCPs could explain the problem and management options in simple terms when the care recipient was present in the outpatients’ appointments and then speak separately to the caregiver, providing more details [61].

#### Initiating conversations during outpatient consultations

There was a lack of consensus with regard to whom caregivers thought should be responsible for initiating conversations about incontinence during dementia related consultations within outpatient settings [61, 62, 64]. Caregivers believed that it was the responsibility of HCPs to initiate conversations about incontinence during both initial consultations and follow-up appointments [61]. However, there were differences depending on whether the care recipient was a parent or a spouse. Caregivers who were daughters or daughters in law would only discuss incontinence with HCPs when it became problematic to manage at home, whereas husbands tended to communicate their wives’ problems much sooner [62]. In contrast, HCPs thought that conversations about incontinence should be initiated by the caregiver [61]. However, when HCPs did initiate conversations about incontinence, they reported that this was appreciated by the caregiver who was receptive and engaging in discussion around the topic [64]. However, within secondary care, not all HCPs saw addressing incontinence as a priority and thought that the topic should be dealt with by the patient’s primary care providers rather than during a specialist secondary care referral [64]. Extended family and friends who were caregivers reported that HCPs do not always ask about incontinence during consultations [62]. A lack of awareness of available resources or concerns about frightening patients/caregivers about potential problems before they occurred was suggested as possible explanations as to why HCPs do not routinely discuss incontinence and fail to initiate conversations about incontinence [64]. Time was found to be the most common barrier reported by HCPs to discussing incontinence, because they believed that a lot of information needed to be covered during the appointments and discussing incontinence issues can take more time than was typically allocated [64]. Possible solutions suggested by HCPs were for the patient/caregiver to have a follow-up appointment to discuss incontinence or to offer referrals to a specialist nurse in continence care [64].

### The language of incontinence during outpatient consultations

Caregivers desired “straight talk” from HCPs about incontinence and its management in relation to PLWD ([61] p. 520). Hispanic caregivers stressed that it was essential for providers to discuss incontinence using language that those with English as a second language could understand. They strongly supported having written materials about incontinence in PLWD and treatment plans available in Spanish [62]. During outpatient consultations, caregivers rarely used the term incontinence, instead use terms such as having accidents, leaking, losing control, wetting or messing their pants, having a urine/bowel problem, urgency, diarrhoea, loose bowels, being unable to hold it, and not getting there in time, difficulty in getting to the bathroom, leaking, and soiling themselves [61, 64]. Health care providers also tend to adopt these terms when discussing incontinence with family caregivers or patients [64]. Caregivers when questioned said that they did not know the right terms and did not want to be disrespectful to their care recipients. However, once they were made aware of the term incontinence, they were happy to use it [61]. Caregivers and HCPs suggested a number of written information resources that could be provided for the caregivers attending outpatient consultations [61, 62, 64] which included the following. A guide for caregivers for use during their spoken interaction with a HCP about continence problems; with definitions of common clinical terms [61]; a pre-visit check list or written materials which facilitated patients/caregivers to indicate whether incontinence was present, which could then prompt the HCP to start a discussion during the consultation [64]; readily available handouts offering more detailed explanations of what had been covered during the appointment [64]; and short, focused handouts that could stand alone and address a single concern [64].

### Theme 3: Delivering individualised continence care

Five of the included studies [72–76] and nine of the non-research publications [10, 33, 77, 81, 84–88] reported findings related to theme 3. Four sub-themes were identified which included the importance of individualised continence care; components of individualised care planning; and HCPs and caregivers working in partnership and establishing a toileting routine within the home environment.

#### The importance of individualised continence care

Targeted and individualised/person-centred continence care [10, 33, 65, 77, 81, 84] that is established after a thorough clinical assessment has taken place [10, 33, 86, 88] is identified as being important. This would include the use of a bladder diary [10]. Individualised continence care is described as care about what is best for the PLWD [10, 80], avoiding harm [10] and about promoting autonomy and independent living [10].

### Components of individualised care plans

A number of different components that may be considered as part of individualised care plans have been identified which include being theory based [33], being concerned with the practical issues [77], and involving multi-components exploring both day time and night care of incontinence care [33]. There was a general consensus that the needs of both PLWD and their caregivers should to be considered [10, 33, 84–86]. The advice given by the Alzheimer’s Society was that a continence care plan should be tailored to the individual. This should aim “to cure toilet problems or incontinence wherever possible” ([78] webpage). Other components to consider include changing medication [77], changes to lifestyle [77], exercise [77], skin care [33, 86], manipulating the type, quantity and timing of food and drink [77], describe support available from HCPs [77], and follow-up advice [77].

Ostaszkiewicz et al. [86] comments that nurses and care workers need support in order to develop individualised strategies to “optimise the care-dependent person’s rest/sleep in the context of the person’s concurrent need for continence and skin care” ([87], p. 524/5)*.* Three studies described individualised care plans as part of wider interventions [74–76]. One was conducted within nursing homes and one member of staff from each home was selected to take part in a training program who then became responsible for educating other staff members. The intervention in this instance was multi-faceted covering individualised and comprehensive care that focused on providing adequate fluids and meals, encouraging patients to use toilets and reducing the size of their continence pads. This approach differed significantly from the usual UI care in which continence pads would be changed only at scheduled times. Improvements across the different methods of urination were observed (continence pads, commode, urinary chamber pot) with only 11% of residents showing improvements during the day which were non-significant, whereas 19% of residents showed significant improvement during the night, changing from using continence pads to using the toilet. Overall, a large number of residents’ toileting remained unchanged following the intervention [74].

Two studies [75, 76] evaluated an environmental skill-building program which was a home environment individualised intervention delivered by occupational therapists, which included toileting and incontinence. The intervention was designed to enhance the caregiver’s ability to problem solve about their environment and to develop effective solutions to situations they considered problematic. The study by Gitlin and Corcoran [75] was a pilot and the 59% of caregivers reported incontinence as problematic in their daily management routine. Problems included night time and/or day time incontinence of the bladder and/or bowel, resistance to toileting, or confusion as to how to perform an aspect of the toileting task. Seventeen effective caregiver initiated environmental solutions for incontinence were observed and of these, 9 solutions (53%) were accepted by the caregivers and integrated into their management routine by visit 5 of the intervention. For the later study by Corcoran and Gitlin [76], 29% of caregivers identified continence as a problem area that needed addressing. Twenty-six attempted strategies that involved assistive devices and of these, 21 (81%) were used. Fifty-one attempted strategies to manipulate the type, quantity, and timing of food and drink and 46 (90%) were used. The authors did not provide any further detail on the nature of the assistive devices.

One further study implemented a person-centred approach that focused on incontinence for residents with cognitive decline in residential treatment facilities [73]. The health workers were provided with training; however, only 20 out of 100 participated although the process outcomes were measured among all residents who agreed to participate in the study. There were no statistically significant mean differences in quality of life scores before and after the intervention or between control and intervention participants. However, the quality of care improved for the intervention participants in that, fewer aids were needed to manage incontinence and an increased number of UI assessments were conducted.

#### Health care professionals and caregivers working in partnership

The importance of HCPs and caregivers working together to deliver individualised/person-centred continence care was a feature of three intervention studies [72, 75, 76] and was encouraged within four pieces of non-research material [77, 81, 84, 85]. Within one intervention study, nurse practitioners worked with the carer to plan the schedule for the PLWD, and this was followed up with monthly phone calls and bi-monthly visits [72]. Occupational therapists worked with the caregivers in a further two intervention studies [75, 76] to deliver solutions to toileting and incontinence problems, which consisted of five visits over 2 [76] or 3 months [75]. Other HCPs that work with PLWD and their caregivers include continence advisors [77] or other HCPs specialising in continence care [81]. Working in partnership with caregivers and PLWD is important [77, 81, 85] and enables HCPs to gather their personal story [84] to work out the best solutions and to ensure that specialist help can be accessed when needed and so that what is recommended is achievable [77].

#### Establishing a toileting routine within the home environment

The importance of developing a regular toileting schedule was discussed briefly within one study [75] and one piece of non-research material [10] and was the focus of one intervention study [72]. The intervention group in the study by Jirovec and Templin [72] were taught an individualised scheduled toileting procedure, which compensated for cognitive impairment by providing these patients with toileting reminders such as verbal prompts. Initially, assignment was to one of two intervention groups: one group of participants was visited every 2 months, and the other group after a 6-month interval. There was also a control group. At the 6-month follow-up, the two intervention groups did not differ with respect to UI, and these two intervention groups were combined to form a single intervention group, plus a control group. The authors conducted a completer’s only analysis and reported that incontinence decreased in the experimental group (28 of the 44 participants still in the study at 6 months) with almost no change in the control group. Further analysis of this data using the non-parametric sign test was conducted, demonstrating a significant decrease of incontinence within the experimental group (Z = −1.83, p < 0.05). The participants were coded according to any decrease in percentage of incontinent episodes versus staying the same or not showing improvement in incontinence. However, two previous reviews conducted a re-analysis of the data which found that although the results favoured the experimental groups, they were not statistically significant [41, 89].

### Overarching synthesis

An overarching summary and a set 26 synthesis summary statements derived from both the descriptive quantitative (that had undergone qualitisation) and qualitative research, and from included policy and guidance documents and four summary statements for the experimental quantitative research, was produced with levels of confidence using the CERQual (see Table 5 and GRADE approaches. Because the design of all the experimental quantitative research were assessed as poorly designed observational studies, the ratings for evidence from each outcome generated using material from these studies were downgraded from ‘low quality’ to ‘very low quality ’[90].

**Table 5:** Overarching synthesis with CERQual and GRADE

**Theme 1: Communication that is dignified, person-centred, and respectful**	
***Communicating in a dignified way***	
1. PLWD and their carers find talking about incontinence distressing and embarrassing CERQual: moderate/studies 2, 3, 4 2. HCPs to build trust and rapport through using humour, having appropriate knowledge and skills by speaking quietly and keeping incontinence issues secret CERQual: moderate/studies 2, 3, 4	
***The attitudes of HCPs towards continence and continence care***	
3. HCPs often ignore toileting requests or avoid routine toileting citing being busy or being uncomfortable with or disinterested in toileting CERQual: moderate/studies 2, 14 4. Staff in acute settings do not consistently promote continence CERQual: very low/study 14 5. HCPs having respect building relationships and using appropriate language CERQual: very low/study 2 6. Interpersonal and communication skills are important and should be a focus of education programs [86, 87] (non-research: ungraded)	
***The importance of non-verbal cues***	
7. PLWD are not always able to recognise and communicate that they need to go to the toilet or indicate that they assistance [10, 80, 81, 83] and they use a variety of non-verbal cues [10, 79, 81–83, 85, 87] CERQual: high: studies 1, 2, 3, 4, 5, 6, 7, 10, 11, 12 and non-research: ungraded 8. HCPs checking PLWD awareness of communication techniques including non-verbal cues through communicating with the family CERQual: moderate/studies 2, 13 9. HCPs being able to recognise the non-verbal signals, body language, facial expressions, behaviours, and signs that PLWD use to communicate that they need to go toilet is crucial [79–81] and this should be a focus education programs for new staff CERQual: moderate/studies 2, 12 and non-research: ungraded	
***Finding the appropriate words and symbols to describe the toilet***	
10. Finding out what words or phrases that PLWD use for describing the toilet is seen as important [79, 81–83] CERQual: very low Study 7 and non-research: ungraded 11. People living with moderate dementia preferred the word toilet compared to those with no cognitive impairments and those with advanced dementia preferred the international symbol for toilet compared to those with mild dementia or no cognitive impairment CERQual: very low/study 7	
***Strategies for improving communication***	
12. HCPs introducing themselves and seeking PLWD approval before performing tasks CERQual: very low/study 4 13. A range of strategies have been identified that include getting to know the PLWD and how they communicate and manage their continence, communicating with the family, prompting, seeing the person has an individual, and checking HCPs communication skills [10, 80–83] CERQual: moderate/studies 4, 13 and non-research: ungraded	
***Using technology to present instructions***	
14. Verbal instructions, presented automatically through simple technology has the potential to be effective in helping persons with mild or moderate AD go to the toilet independently by presenting simple step wise sequential instructions Grade: very low/studies 5, 6	
**Theme 2: Communication during outpatient appointments**	
***Presence of PLWD during outpatient consultations***	
15. Caregivers felt having the PLWD with them during outpatient consultations could cause unnecessary anxiety CERQual: very low/study 1 16. Caregivers felt having the PLWD with them during outpatient consultations would allow greater cooperation with management strategies CERQual: very low/study 1 17. HCPs felt it was important that PLWD were present at appointments CERQual: very low/study 3	
***Initiating conversations during outpatient consultations***	
18. Uncertainty over who should initiate conversations during consultations CERQual: very low/study 3 19. HCPs suggested developing a pre-visit checklist to prompt conversation during consultations CERQual: very low/study 3	
***The language of incontinence during outpatient consultations***	
20. Incontinence and management options are often explained in terms that caregiver find difficult to understand. CERQual: low/studies 1, 3 21. Caregivers and HCPs suggested a variety of written information resources that could be provided CERQual: low/studies 1, 3	
**Theme 3: Delivering individualised continence care**	
***Importance of individualised continence care***	
22. Targeted and individualised/person centred continence care that is established after a thorough assessment has taken place is seen as important [10, 33, 77, 81, 84, 86–88] non-research: ungraded 23. Individualised continence care is about what is best for the PLWD and avoiding harm and about promoting autonomy and independent living [10]. non-research: ungraded	
***Components of individualised care planning***	
24. Individualised care planning should consider the needs of both PLWD and their caregivers and involve multi-components exploring both day time and night care of incontinence are helpful in addressing incontinence in the home care setting [10, 33, 77, 84–86] non-research: ungraded 25. An intervention that involved individualised and comprehensive care for residents in a care home that focused on providing adequate fluids and meal by encouraging patients to use toilets was effective for 19% of residents in reducing the proportion of diapers used Grade: very low/study 9 26. An intervention that involved individual treatment strategies delivered by an occupational therapist and designed to enhance the caregiver's ability to problem solve about their environment. A post-intervention survey reported that this approach enabled caregivers to develop effective solutions to situations they considered problematic which included toileting CERQual: low/studies 10, 11 27. An intervention that involved training health workers in person centred care was effective in improving the quality of care and a reduction in the number of aids needed to manage incontinence GRADE: very low/study 15	
***Health care professionals and caregivers working in partnership***	
28. It is important that HCPs and caregivers work together to deliver individualised/person centred continence care [77, 81, 84, 85] non-research: ungraded	
***Establishing a toileting routine within the home environment***	
29. The importance of developing a regular toileting schedule was highlighted by caregivers [10] CerQUAL: very low/study 10/non-research: ungraded 30. An individualised scheduled toileting program that compensated for cognitive impairment by providing memory-impaired patients with toileting reminders was not shown to have any significant benefits in terms of improving the number of incontinent episodes for PLWD in a home care setting Grade: very low/study 8	

Key: *HCP* health care professional, *PLWD* people living with dementia

#### Communication that is dignified, person-centred, and respectful

People living with dementia and their carers find talking about incontinence distressing and embarrassing (CERQual: moderate [63–65]). Therefore, communicating in a dignified way is important and one way that HCPs feel they can do this is by building trust and rapport through using humour, having appropriate knowledge and skills, and by speaking quietly and keeping incontinence issues secret (CERQual: moderate [63–65]). People living with dementia often report poor attitudes of HCPs towards continence and continence care in that HCPs often ignore toileting requests or avoid routine toileting citing being busy or being uncomfortable with or disinterested in toileting (CERQual: moderate [68] and that staff in acute settings do not consistently promote continence (CERQual: very low [66]). What PLWD report to be helpful is HCPs having respect, building relationships and using appropriate language (CERQual: very low [63]). Interpersonal and communication skills are important and should be a focus of education programs (non-research: ungraded [86, 87]). Being able to recognise verbal cues is important because PLWD are not always able to recognise and communicate that they need to go to the toilet or indicate that they need assistance and they use a variety of non-verbal cues (CERQual: high [61–63, 65, 67, 68, 70, 71, 75, 76]; and non-research: ungraded [10, 79, 81–83, 85, 87]) as well as HCPs checking PLWD awareness of communication techniques including non-verbal cues through communicating with the family (CERQual: moderate [63, 69]). The ability of HCPs to recognise non-verbal signals, body language, facial expressions, behaviours, and signs that PLWD use to communicate that they need to go toilet is crucial (non-research: ungraded [79–81]) and should be a focus within education programs for new staff (CERQual: low [63, 68]). Finding out what words or phrases PLWD use for describing the toilet as well as being able to recognise familiar gestures is also seen as important (CERQual: very low [67]); non-research: ungraded [67, 79, 81–83]. It has been demonstrated that PLWD preferred the word toilet compared to those assessed with no cognitive impairment and those with advanced dementia preferred the international symbol for toilet compared to those assessed with mild dementia or with no cognitive impairment (CERQual: very low [67]). A range of strategies have been identified that include getting to know the PLWD and how they communicate and manage their continence, communicating with the family, prompting, seeing the person has an individual, and checking HCPs communication skills study (CerQUAL: moderate [65, 69]; non-research: ungraded [10, 80–83]) as well as HCPs introducing themselves and seeking PLWD approval before performing tasks (CERQual: very Low [65]). Verbal instructions, presented automatically through simple technology, has the potential to be effective in helping persons assessed with mild or moderate AD go to the toilet independently by presenting simple step wise sequential instructions (GRADE: very low [70, 71]).

#### Communication during outpatient appointments

Health care professionals felt that it was important that PLWD were present with their caregivers during outpatient appointments; however, caregivers felt that although this would allow greater cooperation with management strategies, this would also cause unnecessary anxiety (CERQual: very low [61, 62, 64]). There was uncertainty over who should initiate conversations during consultations (CERQual: very low [64]), and HCPs suggested developing a pre-visit checklist to prompt conversation during consultations (CERQual: very low [64]). Caregivers reported that incontinence and management options are often explained in terms they find difficult to understand (CERQual: low [61, 62, 64]), and both caregivers and HCPs suggested a number written information resources that could be provided for the caregivers attending outpatient consultations to ameliorate this problem (CERQual: low [61, 62, 64]).

#### Delivering individualised continence care

Targeted and individualised/person-centred continence care that is established after a thorough assessment has taken place is seen as important (non-research: ungraded [10, 33, 77, 81, 84, 86–88] and is about what is best for the PLWD and avoiding harm and about promoting autonomy and independent living (non-research: ungraded [10]). Individualised care planning should consider the needs of both PLWD and their caregivers and involve multi-components exploring both day and night time incontinence care, these are helpful in addressing incontinence in the home care setting (non-research: ungraded [10, 33, 77, 84–86]). It has been identified as imperative that HCPs and caregivers work together to deliver individualised/person-centred continence care (non-research: ungraded [77, 81, 84, 85]. An intervention that involved individualised and comprehensive care for residents within a care home focused on providing adequate fluids and meal by encouraging patients to use toilets was effective for 19% of residents in reducing the proportion of continence pads used (GRADE: very low [74]). Another intervention that involved training health workers in person-centred care was effective in improving the quality of care and a reduction in the number of aids needed to manage incontinence (GRADE: very low [73]). A post-intervention survey reported an intervention that involved individual treatment strategies delivered by an occupational therapist and was designed to enhance the caregiver’s ability to problem solve about their environment enabled caregivers to develop effective solutions to situations they considered problematic which included toileting (CERQual: low [75, 76]). The importance of developing a regular toileting schedule was highlighted by caregivers CERQual: very low [75]; Non research: ungraded [37]). However, an individualised scheduled toileting program that compensated for cognitive impairment by providing those with cognitive impairment with toileting reminders was not shown to have any significant benefits in terms of reducing the number of incontinent episodes for PLWD in a home care setting GRADE: very low [72].

## Discussion

Maintaining continence has been highlighted as a major issue for patients with long-term conditions, which includes PLWD, and understanding the best ways to support continence and the management of incontinence in PLWD has been recognised as a research priority [91]. This review is therefore timely and offers up a summary of the available knowledge to date that stakeholders and those caring for PLWD identified as important; key issues were communication and individualised care planning.

The first overarching synthesis highlighted with a high level of confidence that PLWD are not always able to recognise that they have continence needs, need to go to the toilet, or verbally communicate that they need assistance. The wider literature acknowledges that patients with long-term conditions including PLWD can maintain continence with assistance, but that the reality is often that many are unnecessarily treated as incontinent in hospital and care home settings [91]. This synthesis identified that continence care is often considered a low priority by some healthcare staff and that they are sometimes unable to recognise when PLWD have continence needs unless this is verbally communicated. It was also highlighted that a variety of non-verbal cues are often used by PLWD to indicate their continence needs and that this can be further facilitated when HCPs familiarise themselves with these words, phrases, and non-verbal signals such as facial expressions, familiar gestures, behaviours, or signs, that each PLWD uses to communicate this need. There is very low-quality evidence suggesting that some staff do not appear to consistently promote continence and in some instances were too busy or disinterested to support individual continence care in acute settings. Although a range of communication strategies have been suggested within the non-research literature, what is needed is a renewed focus on improving both verbal and non-verbal communication strategies and recognition, so that distress associated with the use of language and embarrassment around maintaining continence for PLWD can be minimised. To facilitate continence care across all settings, it is important that training of those who work with PLWD and their carers should include continence care that also incorporates the skills of interpersonal communication and recognition. A recent collaborative workshop addressing the need for continence research also highlighted that there is currently a lack of training for health and social care professionals in continence care [91].

Although some evidence maps across these themes, there are important gaps between what caregivers and HCPs have identified as deficiencies in continence care for PLWD when considering communication, and a lack of robustly evaluated interventions which attempt to address these deficiencies. This evidence gap is reflective of the wider evidence base to support the quality of dementia care and communication. For example, Machiels et al. reported that only a few intervention studies have been concerned with examining how communication between nursing staff and PLWD can be improved [92] and Eggenberg et al. found no studies which looked at identifying ways to improve communication between physicians and PLWD [93]. More research examining communication is needed to support effective care. A review identified that when training in communication skills has been conducted with professional and family caregivers, then the quality of life and wellbeing of PLWD in both nursing homes and home-care settings improved, which in turn increased positive interactions [93].

The second overarching synthesis finding comprised very low-quality evidence that explored different aspects of communication occurring between PLWD, caregivers, and HCPs during outpatient appointments. The caregivers’ role has been characterised as one of both an informant and an advocate during an outpatient appointment [94]. The synthesis identified that HCPs feel it is important that PLWD were present at outpatient appointments; however, caregivers expressed divided opinions as to whether PLWD should attend outpatient appointments with them. Previous research on doctor-patient communication has focused on disclosing a diagnosis of dementia [94] and caregivers report discomfort in the presence of the PLWD when divulging sensitive information [95]. An educational intervention that sought to improve patient-centred care for PLWD and their carers during medical encounters with old age psychiatrists suggested a number of changes to the consultation structure [96]. One of which was to offer the PLWD and carer a choice of whether they attended the consultation separately as well as together in order than patient-centred care during their consultations could be developed. We did not find evidence of any strategies for use in outpatient settings to better support PLWD, their families, or staff, about incontinence or promoting continence. The on-going care of PLWD in both outpatient and primary care setting when managing continence is an area that requires further research.

The final overarching synthesis was concerned with the delivery of individualised continence care. It is well-documented in a number of guidelines and across the material produced by various charities, that individualised care plans should consider the needs of both PLWD and their caregivers, and involve multi-components exploring both day and night time care of incontinence if they are to be helpful in facilitating continence. What this synthesis revealed is that there is a lack of interventions related to the delivery of individualised continence care for PLWD with only three intervention studies [72, 75, 76] providing very low quality evidence that incorporated some aspect of individualised care. Hagglund in her systematic review of incontinence care for PLWD also reported a lack of evidence-based interventions and pointed out the need for “effective continence-promoting interventions and improved individualised nursing” ([40] p. 311). There is evidence also of a ‘policy/practice-research gap’, given there is a significant and well-meaning focus on person-centred care in policy and practice yet there is little research to support staff in how to achieve this in practice. It is also recognised as important that HCPs and caregivers work together to deliver individualised/person centred continence care for PLWD. Each PLWD is unique and HCPs need to be enabled to recognise the specific individual needs of each person as opposed to assuming a ‘one size fits all’ approach when it comes to continence care. Continence care needs to be personalised and responsive to the PLWD preferences and needs.

## Limitations

The search was for English language-only materials. The studies included in this review varied in methodological quality, which impacts on the overall results and conclusions that can be drawn. A strength of this review has been the inclusion of the views and interests of stakeholders, including PLWD and family carers which led us to focus on communication and individualised care. Another strength of this review was the use of the CERQual approach which allowed us to determine a level of confidence in the synthesised review findings.

## Conclusions

The findings from the syntheses derived from this review of the international literature can help inform innovations in continence care for PLWD in the acute hospital setting. Recognising that PLWD are not always able to verbally communicate their continence needs or that they require assistance is important. Incorporating interpersonal and communication skills in the context of continence care for those working with PLWD is crucial for continence to be supported and maintained in the acute setting. Training of those who work with PLWD and their carers should include continence care and incorporate interpersonal and communication skills.

Developing and implementing interventions that seek to improve the delivery of individualised continence care within the acute setting that can be tried and tested and could be ‘rolled out’ to suit the majority of PLWD and their caregivers would be difficult. Taking into account the varying and many needs of individual people, their circumstances and symptoms would make such interventions challenging. The complexity of living with continence problems alongside any other long-term health conditions such as dementia has been acknowledged; however, addressing this requires a holistic approach [91]. What we do know is that continence care in the acute setting, which is tailored to the individual and that is developed in a partnership between HCPs and caregivers is more likely to be successful.

## Supplementary Information

**Additional file 1: S1.** PRIMSA flow diagram for mapping

**Additional file 2: S2.** Search strategy for Medline

**Additional file 3: S3.** Included non-research material and extracted data

**Additional file 4: S4.** Studies excluded after full text screening

## Abbreviations

AD: Alzheimer’s disease
CERQual: Confidence in the Evidence from Reviews of Qualitative research
FI: Faecal incontinence
GRADE: Grading of Recommendations Assessment, Development, and Evaluation
HCP: Health care professionals / providers
LTCF: Long-term care facility
PLWD: People living with dementia
RCT: Randomised controlled trials
UI: Urinary incontinence
