# Supplementary material for: Understanding how to facilitate continence for people with dementia in acute hospital settings: a mixed methods systematic review and thematic synthesis
Source: Syst Rev. 2021 Jul 6;10:199. doi: 10.1186/s13643-021-01743-0 (PMC8262033; doi:10.1186/s13643-021-01743-0)
Supplement: Supplementary file 1 — Additional file 1: S1. PRIMSA flow diagram for mapping [file 13643_2021_1743_MOESM1_ESM.docx]

**Additional File S1: PRSIMA flow diagram for mapping**

Back chaining of review papers

(35 citations)

Database searching

Cinahl (1,169 citations)

Medline (144 citations)

Duplicates removed

(16 citations)

Screening titles and abstracts of remainder

(1,332 citations)

Screening of full papers

(114 citations)

Included papers

Primary research papers (40 studies across 48 citations)

General discussion/opinion papers (17)

Reviews (13 reviews across 17 citations)

Audits (2)

Guidelines (2)

Documentary analysis (1)
