# Supplementary material for: Understanding how to facilitate continence for people with dementia in acute hospital settings: a mixed methods systematic review and thematic synthesis
Source: Syst Rev. 2021 Jul 6;10:199. doi: 10.1186/s13643-021-01743-0 (PMC8262033; doi:10.1186/s13643-021-01743-0)
Supplement: Supplementary file 2 — Additional file 2: S2. Search strategy for Medline [file 13643_2021_1743_MOESM2_ESM.docx]

**Addiotnal File S1: Search strategy for medline**

**Ovid MEDLINE(R) ALL: communication** (Inception to August 2020)

1 dement*.mp.

2 alzheimer*.mp.

3 exp Dementia/ )

4 ((cognit* or memory* or mental*) adj3 (declin* or impair* or los* or deteriorat*)).mp.

5 exp DEMENTIA, MULTI-INFARCT/

6 exp FRONTOTEMPORAL DEMENTIA/

7 exp DEMENTIA, VASCULAR/

8 exp senile dementia/

9 exp Alzheimer Disease/

10 exp Cognition Disorders/

11 exp mild cognitive impairment/

12 ("limited cognitive disturbance*" or "mild cognitive disorder*").mp.

13 1 or 2 or 3 or 4 or 5 or 6 or 7 or 8 or 9 or 10 or 11 or 12

14 exp urinary incontinence/

15 (incontinen$ or continen$).tw.

16 exp fecal incontinence/

17 ((fecal or faecal) adj2 (incontinen$ or continen$)).tw.

18 (stool$ adj2 (incontinen$ or continen$)).tw.

19 (Conservative adj2 (intervention$ or measure$)).ti,ab.

20 (Continence adj2 restoration).ti,ab.

21 (Continence adj2 care).ti,ab.

22 ((UI or Incontinence) adj2 (care or manag$ or reduc$ or assess$ or contain$)).tw.

23 toilet training/

24 toilet$.tw.

25 14 or 15 or 16 or 17 or 18 or 19 or 20 or 21 or 22 or 23 or 24

26 exp NONVERBAL COMMUNICATION/ or exp COMMUNICATION/

27 (cues or behavio?r* or word* or signs* or signage or promp* or reassur* or speak* or reinforc* or language or visual or language or expression* or voice).ti,ab.

28 (discour* or dialog* or disclos* or intera* or communica* or talk* or speak* or verbal or non-verbal or interpersonal or convers*or relation*).ti,ab.

29 26 or 27 or 28

30 13 and 25 and 29

31 limit 30 to English language

**Ovid MEDLINE(R) ALL: Individualised care plans (Inception to August 2020)**

1 dement*.mp.

2 alzheimer*.mp.

3 exp Dementia/

4 ((cognit* or memory* or mental*) adj3 (declin* or impair* or los* or deteriorat*)).mp.

5 exp DEMENTIA, MULTI-INFARCT/

6 exp FRONTOTEMPORAL DEMENTIA/

7 exp DEMENTIA, VASCULAR/

8 exp senile dementia/

9 exp Alzheimer Disease/ (

10 exp Cognition Disorders/

11 exp mild cognitive impairment/

12 ("limited cognitive disturbance*" or "mild cognitive disorder*").mp.

13 1 or 2 or 3 or 4 or 5 or 6 or 7 or 8 or 9 or 10 or 11 or 12

14 exp urinary incontinence/

15 (incontinen$ or continen$).tw.

16 exp fecal incontinence/

17 ((fecal or faecal) adj2 (incontinen$ or continen$)).tw.

18 (stool$ adj2 (incontinen$ or continen$)).tw.

19 (Conservative adj2 (intervention$ or measure$)).ti,ab.

20 (Continence adj2 restoration).ti,ab.

21 (Continence adj2 care).ti,ab.

22 ((UI or Incontinence) adj2 (care or manag$ or reduc$ or assess$ or contain$)).tw.

23 toilet training/

24 toilet$.tw.

25 14 or 15 or 16 or 17 or 18 or 19 or 20 or 21 or 22 or 23 or 24

26 exp Patient Care Planning/

27 (Individual* adj3 plan*).tw.

28 (Personal* adj3 plan*).tw.

29 26 or 27 or 28

30 13 and 25 and 29

31 limit 30 to English language
