# Supplementary material for: Understanding how to facilitate continence for people with dementia in acute hospital settings: a mixed methods systematic review and thematic synthesis
Source: Syst Rev. 2021 Jul 6;10:199. doi: 10.1186/s13643-021-01743-0 (PMC8262033; doi:10.1186/s13643-021-01743-0)
Supplement: Supplementary file 3 — Additional file 3: S3. Included non-research material and extracted data [file 13643_2021_1743_MOESM3_ESM.docx]

**Additional File S3: Studies excluded after full text screening**

1. Burkhard et al 2018: Clinical guideline for the European association of Urology: Urinary incontinence.

*Reasons for exclusion:* No data on for people living with dementia, Alzheimer’s disease or cognitive impairment within the guideline

1. Billing et al (2009): Privacy and Dignity in continence care project

*Reasons for exclusion*: Sample was people over 65 years of age who had the cognitive and linguistic ability to participate

1. National Institute for Health and Care Excellence (2014): Faecal incontinence in adults. Clinical guideline 49

*Reasons for exclusion:* No data on communication of individualised care plans in the section relevant for people living with dementia

1. Royal College of Psychiatrists: National audit of dementia care in general hospitals 2016-2017. Third round of audit report

Reasons for exclusion: Continence only mentioned in relation to assessment

1. Royal College of Physicians 2010: National audit of continence care

Reasons for exclusion: No data on for people living with dementia, Alzheimer’s disease or cognitive impairment within the guideline

1. Albert 1999: The caregiver as part of the dementia management team

*Reasons for exclusion:* Not about toileting or incontinence

1. Allwood et al 2017: Should I stay or should I go? How healthcare professionals close encounters with people with dementia in the acute hospital setting

*Reasons for exclusion:* Not about toileting or incontinence

1. Burgener et al 1992: Caregiver and environmental variables related to difficult behaviors in institutionalized, demented elderly persons

*Reasons for exclusion:* Not about communication in relation to toileting

1. DiZazzo et al 2014: Addressing everyday challenges: feasibility of a family caregiver training program for people with dementia

*Reasons for exclusion*: Research covers communication in relation to nutrition and transfer and toileting but not about communication in relation to toileting

1. Ghatak 2011: A unique support model for dementia patients and their families in a tertiary hospital setting: description and preliminary data

*Reasons for exclusion:* Support program for dementia carers and not about communication in relating to toileting

1. Lanciono et al 2013: Supporting daily activities and indoor travel of persons with moderate Alzheimer's disease through standard technology resources

*Reasons for exclusion:* Not about communication in relation to toileting

1. Moyle et al 2016: They rush you and push you too much ... and you can't really get any good response off them': A qualitative examination of family involvement in care of people with dementia in acute care

*Reasons for exclusion:* Not about communication in relation to toileting

1. Norbergh et al 2001: How patients with dementia spend their time in a psycho-geriatric unit

*Reasons for exclusion:* Not about communication in relation to toileting

1. Panella 1986: Toileting strategies in day care programs for dementia

*Reasons for exclusion*: Discussion article

1. Perilli et al 2013: Video prompting versus other instruction strategies for persons with Alzheimer's disease

*Reasons for exclusion:* Not about communication in relation to toileting

1. Tales et al 2017: Dementia-friendly public toilets

*Reasons for exclusion:* Correspondence piece

1. Uchimoto et al 2013: Investigation of toilet activities in elderly patients with dementia from the viewpoint of motivation and self-awareness

*Reasons for exclusion:* Not about communication in relation to toileting

1. Warkentin 1992: Implementation of a urinary continence program

*Reasons for exclusion*: Not about communication in relation to toileting

1. Williams et al 1995: Patients with dementia and their caregivers 3 years after diagnosis. A longitudinal study

*Reasons for exclusion:* Not about communication in relation to toileting

1. Svedas and Wise 2012: Improving bowel care in residential aged care facilities

*Reasons for exclusion:* Not about individualised care plans and continence care

1. Bucci 2007: Be a continence champion: Use the CHAMP tool to individualise the plan of care

*Reasons for exclusion:* Discussion article

1. Rogers et al 1999: Improving bowel care in residential aged care facilities

*Reasons for exclusion*: Not about individualised care plans and continence care

1. Corcoran et al 2002: An occupational therapy home-based intervention to address dementia-related problems identified by family caregivers

*Reasons for exclusion*: No data regarding individualised care plans and continence care

1. Prizer and Zimmerman 2018: Progressive support for activities of daily living for persons living with dementia

*Reasons for exclusion*: Not about individualised care plans and continence care

1. Drennan et al 2017: Meeting the needs of older people living at home with dementia who have problems with continence

*Reasons for exclusion*: Not about individualised care plans and continence care

1. Olthof-Nefke et al 2018: Improving communication between persons with mild dementia and their caregivers: Qualitative analysis of a practice-based logopaedic intervention

*Reasons for exclusion:* Not about communication in relation to toileting

1. Soderman et al 2018: Caring and uncaring encounters between assistant nurses and immigrants with dementia symptoms in two group homes in Sweden-an observational study

*Reasons for exclusion:* Not about communication in relation to toileting

1. Dahlke et al 2019: The educational needs of nursing staff when working with hospitalised older people

*Reasons for exclusion:*  Not about the care of people living with dementia

1. Foster et al 2019: Patient-centred care training needs of health care assistants who provide care for people with dementia

*Reasons for exclusion:* Not about toileting or incontinence

1. Samuelsson et al 2019: Digital communication support in interaction involving people with dementia

*Reasons for exclusion:* Not about communication in relation to toileting

1. Stanyon et al 2019: Effects of care assistant communication style on communicative behaviours of residents with dementia: a systematic multiple case study

*Reasons for exclusion:* Not about communication in relation to toileting

1. Mariana et al 2018: The impact of a shared decision-making training program on dementia care planning in long-term care

*Reasons for exclusion:* Not about toileting or incontinence

1. Villar et al 2018: Involving institutionalised people with dementia in their care‐planning meetings: lessons learnt by the staff

*Reasons for exclusion:* Not about toileting or incontinence

1. Yenisehir et al 2019: Knowledge and practice of nursing home caregivers about urinary incontinence

*Reasons for exclusion:* Not about toileting or incontinence

1. Wijk et al 2018: Person-centered incontinence care in residential care facilities for older adults with cognitive decline: Feasibility and preliminary effects on quality of life and quality of care

*Reasons for exclusion:* Not about toileting or incontinence
