# Supplementary material for: Understanding how to facilitate continence for people with dementia in acute hospital settings: a mixed methods systematic review and thematic synthesis
Source: Syst Rev. 2021 Jul 6;10:199. doi: 10.1186/s13643-021-01743-0 (PMC8262033; doi:10.1186/s13643-021-01743-0)
Supplement: Supplementary file 4 — Additional file 4: S4. Studies excluded after full text screening [file 13643_2021_1743_MOESM4_ESM.docx]

**Additional file S4_Included non research material and extracted data**

| **Document details** | **Extracted material relevant to the review** |
| --- | --- |
| Web page  1. Alzheimer’s Society 2016 [78]  [Managing toilet problems and incontinence](http://www.alzheimers.org.uk/site/scripts/documents_info.php?documentID=136)’, Factsheet 502LP  Alzheimer’s Society, London, UK | **Individualised management plans**  *“After a thorough assessment the continence adviser will write up a continence care plan* ***tailored to the individual****. This should include things that the person with dementia and any carer can do to help. It should also describe the support that professionals should provide, as well as follow-up and next steps”.*  *“The aim should be to cure toilet problems or incontinence wherever possible. This should be agreed with the person with dementia and their carer. In many cases, identifying and addressing practical issues, changing medications or making simple changes to lifestyle (such as diet, drinks and exercise) can help to achieve this”.*  Webpage pg no unavailable |
| Book  2. Alzheimer’s Society and Gray 2014 [79]  Support and care for people with dementia at home: A guide for homecare workers  Alzheimer's Society, London, UK | **Communication**  ***“Pacing*** *may indicate that a person wants to use the toilet”*  Ebook pg no unavailable |
| Webpage  3. Dementia UK 2017 [80]  Continence  Dementia UK, London, UK | **Communication**  *“If possible, ask the person how you can help them manage their continence. Find out about previous routines or habits e.g. frequency of bowel movements*  *Look for* ***non-verbal signs*** *that the person might need the toilet, such as fidgeting, pacing, holding their crotch or their stomach, or going to the corner of the room*  *Pay attention to the language the person uses, such as saying phrases like they ‘need to go out’”*  Webpage pg no unavailable |
| Web page  4. Social care Institute for Excellence 2015 [81]  When people with dementia experience problems related to using the toilet  Social care Institute for Excellence, London, UK | **Communication**  ***Picking up clues***  *"The better we know the people with dementia we care for, the easier it is to support their continence. For example, we may discover that one person usually needs to go to the toilet about half an hour after breakfast. Providing a discreet prompt 20 minutes after breakfast – just before the need is likely to arise – will help to ensure that the person gets there in time. Someone who has always hated public toilets may prefer a private individual toilet rather than a cubicle in a row of toilets”.*  Webpage pg no unavailable  *“If a person is not able to tell us in words that they need to go to the toilet, we’ll have to get to know the* ***non-verbal signals*** *that the person uses to communicate this. It may be, for example, that someone starts pulling at their clothing when they need to go to the toilet. Someone else might make a particular sound”.*  Webpage pg no unavailable |
| Guideline  5. Alzheimer’s Europe 2014 [10]  Guidelines on continence care for people with dementia living at home  Alzheimer’s Europe, Luzembourg | **Individualised management plans**  *“Continence care and treatment should be provided in accordance with the needs of carers and people with dementia The aim is to try to do what is best for the person with dementia and avoid doing harm, This must take into consideration their individuality, promote autonomy, wellbeing and independent living through the provision of appropriate and* ***individualised assessment and care****”.*  Pg 29  *“To* ***tailor assistance to individual needs****, it may be helpful for the person with dementia (with the help of a carer if necessary) to keep a bladder diary”.*  Pg 39  *“Timed voiding: This is where the person is toileted regularly by their carer for example twice hourly and voiding is recorded. The time between voiding is fixed during the training. A voiding schedule can then be charted to match the* ***individual’s voiding pattern****”*  Pg 39  *“Habit retraining: This involves identifying the person’s natural voiding pattern and with the help of a carer developing an* ***individualized toileting schedule*** *to prevent accidents from occurring..”*  Pg 39  **Communication**  *‘Measures to* ***facilitate communication*** *and support decision-making*  *Speak slowly and clearly but without exaggerating.*  *Maintain eye contact.*  *Find a quiet place with no distractions for the discussion.*  *Adapt your language to the perceived level of understanding of the person with dementia.*  *Give the person enough time to respond.*  *Ask one question at a time.*  *Avoid overloading the person with too much information at once. Help the person to express what is currently important to them.*  *Feed information back to check that you have understood and to remind the person with dementia what has been said so far.*  *Use any relevant materials (e.g. objects, brochures and notes).*  *Look out for signs of fatigue, confusion or lack of concentration.*  *Provide reassurance, if needed, through appropriate touch, body language and tone of voice’*  Pg 32  *‘Try to recognise and assess signs that the person with dementia may need to go to the toilet as perhaps s/he is unable to* ***communicate*** *the need or is not sufficiently aware of it and prompt the person to use the toilet using appropriate* ***communication skills****. Such signs might include a different posture, pulling at clothes, anxiety, restlessness, fidgeting or a worried expression. However, it is important to consider the possibility that some of these signs might for some people be an expression of pain of psychological distress’.*  Pg 38 |
| Guideline  6. National Collaborating Centre for Mental Health (updated 2007) [80]  A NICE-SCIE guideline on supporting people with dementia and their carers in health and social care  Clinical guideline [CG42]  The British Psychological Society & The Royal College of Psychiatrists, Leicester, UK | **Communication**  ***“Communication strategies*** *should be considered to find the best way to prompt the person to use the toilet at regular intervals or at moments when the person indicates need. Familiar phrases or words should be explored or gestures or pictures used.”*  Pg 170 |
| Report  7. Thompson et al., 2013 [82]  [Dementia: Commitment to the care of people with dementia in hospital settings](https://www.rcn.org.uk/-/media/royal-college-of-nursing/documents/publications/2013/january/pub-004235.pdf)  Royal College of Nursing, London, UK | **Individualised management plans**  “Principle 4:  *Care plans will be person-centred, responsive to individual needs and support nutrition, dignity, comfort, continence, rehabilitation, activity and palliative care*  *This will be supported by:*  *1) routine gathering of personal life story information*  *2) involvement of family and friends in care planning.”*  Pg 21 |
| Guidance  8. Potter 2015 [83]  Excellence in continence care;  Practical guidance for commissioners, providers, health and social care staff and information for the public  NHS England, Reading, UK | **Individualised management plans**  *“Personalised care and support planning is the key vehicle by which staff work together with people and carers to meet individual care needs, supporting those with complex care needs identified through case finding and risk stratification.”*  Pg 10 |
| Report  9. Care Quality Commission 2014 [81]  Cracks in the pathway.  People’s experiences of dementia car as they move between care homes and hospitals  Care Quality Commission, Gallowgate, UK | **Communication**  *4.2.6 Privacy and dignity*  *Everybody has the right to be shown respect and treated with compassion. For people living with dementia this is particularly important in helping to recognise them as individuals, and acknowledging their difficulties in being able to express their wishes. Understanding the individual and recognising the behaviours that they may use to express their feelings is also important. Where staff had good knowledge of the people they cared for they were respectful and had built good relationships with them*  *Quote “During our observations we saw that staff were very responsive to people’s needs and were able to recognise non-verbal signals which indicated that people required support. For example, we observed one person became a little agitated while sat in their chair [and] staff recognised this as a sign the person may need the toilet. They asked the person if this was the case and supported them appropriately.”*  *Respect for someone’s dignity is not only demonstrated through behaviours, but also through the use of language and the care environment. We saw examples where language used was not respectful. A family member told us about their experience of disrespectful care for their relative*  *Quote “Lack of awareness around language used. Seeing behaviour as uncooperative rather than merely anxious and confused. Using term ‘dirty’ around incontinence to the resident. Patronising behaviour on occasion.”*  *Pg 25* |
| Web based booklet  10. Care Inspectorate 2015[82]  Promoting continence for people living with dementia and long term conditions  Care Inspectorate, UK | **Individualised management plans & Communication**  *“Give me the opportunity to go to the toilet at times that are ‘normal’ for me*  *Work with me and other people to find the best solution, and access specialist help when needed. Pads are not the only option.*  *Ask me about my normal bowel/bladder habits, routines and lifestyle*  *Involve my family, carers and friends in finding out about me and my culture but remember I have a voice too”*  *Know me and how I communicate*  *Listen carefully to any words I use, to help you get to know what I am trying to tell you.*  *If I can’t tell you clearly what I need, pay close attention to my body language, facial expression, behaviour and any signs I may use.*  *Don’t assume anything. Hear me, see me and listen to me”* |
| Framework  11. Ostaszkiewicz et al., 2017 [84]  Dignity in Continence Care Framework  Australia | **Communication**  “*The Dignity in Continence Care Framework additionally champions therapeutic communication that aims to build the care dependent person’s resilience, and enhance their physiological and psychological wellbeing. Addressing the continence care needs of another person in a holistic manner requires not only scientific knowledge, such as knowledge of the etiology of incontinence; it also requires the application of highly developed interpersonal and therapeutic communication skills to manage the interpersonal encounter in ways that protect the person’s integrity. Incontinence is a highly stigmatized condition that elicits profound emotions”.*  pg 523  “Communicating therapeutically about incontinence with any person, including people with dementia, involves the demonstration of warmth, compassion and humanity”.  pg 523  “Future education programs about providing continence care for care-dependent people should include role plays that equip students with the knowledge and skills to communicate therapeutically”.  pg 523  **Individualised care**  *“The goal of a foundational continence assessment is to help those involved in caring for care-dependent older people to identify*  *Targeted and individualized continence care”.*  pg 524  *“Education programs about incontinence are required to equip nurses and care workers with the knowledge and skills to conduct a foundational continence assessment. In order to identify and act upon bladder and bowel signs and symptoms that warrant further attention, this education should address normal and abnormal bladder/bowel function. Arguably, this education should also address methods to non-intrusively collect information about the frequency and severity of a care-dependent person’s bladder and bowel symptoms. Consistent with this education, is the need to support nurses and care workers to develop individualized strategies to optimize the care-dependent person’s rest/sleep in the context of the person’s concurrent need for continence and skin care”.*  pg 524/45 |
| Model  12. Ostaszkiewicz et al., 2018 [85]  Model of Attributes to Abuse of Dependent Elders in Continence Care” (MADE-CC)  Ostaszkiewicz et al, Australia | **Communication**  *“Coercive continence care practices include the use of verbal or physical force to wash a person, to accept wearing incontinence pads or other forms of incontinence containment and to accept continence checks. The underlying intent is to ensure a care recipient is clean and to protect them from visible incontinence that is to ensure their incontinence is contained and concealed”.*  pg 2  “*Chastising a person for being incontinent involves admonishing them and arguably represents a form of verbal abuse. It causes embarrassment and shame. The anticipation or experience of being chastised or admonished for being incontinent is likely to function as a powerful form of social conditioning that affects how individuals respond to attempts to assist them with their incontinence. It is also possible that some carers use chastisement because this is how they learnt to communicate about incontinence in early childhood”.*  pg 2  *“To minimise the potential for harmful caregiving associated with continence care, formal and family carers need education about therapeutic communication strategies that build a care recipient’s resilience and promote their physiological and psychological well-being in the caregiving encounter. This education should also help carers develop an empathetic understanding about basic human emotional responses to incontinence and care dependence and to recognise the effect of these emotions on their own behaviours, and those of the care recipient”.*  pg 8 |
| Guideline  13. Abrams et al, 2017 [33]  6th International Consultation on Incontinence  International Continence Society, Bristol, UK | **Individualised care**  *“The general guidelines should apply for choosing the best management of incontinence in AD patients. The treatment should be tailored to individual patient needs and disease status”.*  pg 908  **Individualised care (frail elderly)**  *“Interventions or approaches to caring for an individual with UI and cognitive impairment need to be* *tailored to the person’s unique abilities and disabilities”.*  Pg 1040  *“Interventions for UI should be theory-based, multi-component, interdisciplinary and person-centred”.*  pg 1040  *Interventions To Manage Night-Time Incontinence In Long-Term Care*  *“The combined findings of these trials suggest that continence care at night in long-term care settings can, and should be individualized based on an assessment of residents’ skin health; their ability to spontaneously move in bed; and on their sleep/ wake status”.*  pg 1039 |
| Information sheet  14. Alzheimer Scotland 2009 [86]  Continence management – advice for carers of people with dementia  Alzheimer Scotland, UK | **Communication**  *Observing behaviour*  *• If the person is fidgeting, getting up or down or pulling at clothes it may be because he or she wants to go to the toilet.*  Pg 3 |
